# When something goes wrong in medical care

**Published:** 2019-09-10

**Authors:** David Yorston

**Affiliations:** 1 Consultant Ophthalmologist: Tennent Institute of Ophthalmology, Gartnavel Hospital, Glasgow, Scotland, UK.

**As health workers, we do everything within our power to ensure that our patients have the best visual and clinical outcomes possible. What should we do if something goes wrong?**

**Figure:**
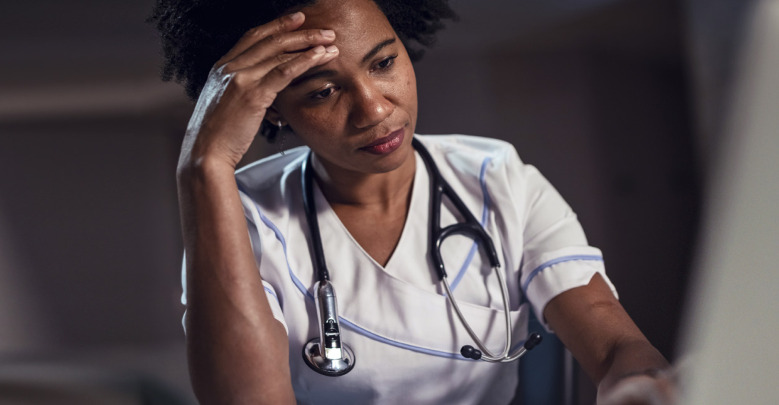
Being involved in a medical error can be devastating; health workers will need the support of senior colleagues (p. 26). STOCK IMAGE

Health care is an inherently dangerous activity. We give people drugs that can be poisonous and use sharp instruments in their eyes. Sometimes, these activities will have harmful consequences. What should we do when someone comes to harm as a result of something we have, or have not, done?

The UK's General Medical Council – the body that sets ethical standards for doctors in the UK – provides guidance^1^ that emphasises the duty of candour: the duty to be open and truthful with our patients. If something goes wrong, we have to tell our patients, give them a full explanation, and apologise.

The guidance emphasises that the explanation and apology should be delivered by a senior clinician. That person may not be at fault, but she or he is responsible for the care of the patient. A more senior health worker may also be more likely to have the knowledge and experience needed in order to answer the patient's questions.

If the apology and explanation is to be delivered by a senior clinician, the more junior members of the team have a duty to inform her or his senior colleague about the error. This can be an uncomfortable moment; therefore, senior staff have a duty to encourage a culture of reporting errors without fear of retribution.

In this type of environment, most errors will be acknowledged as a system failure in which a combination of circumstances have caused an error. Occasionally, a mistake by one individual will be the main cause of the event. In both situations, the default response should be to investigate the cause of the error and provide education and training where needed, rather than taking disciplinary action. This should be reserved for the very rare cases in which a health care worker is deliberately negligent.

## When to tell the patient

Sometimes, patients may not be aware that anything has gone wrong (for example, if a complication occurs while the patient is anaesthetised). In this situation, you may feel that no useful purpose is served by informing the patient of the adverse event. However, failure to inform the patient is dishonest. The complication may come to light if the patient is examined by another doctor, in which case your original error is compounded by your lack of honesty.

It may also be tempting to wait until the final outcome of the event is known. However, we have a duty to provide a full explanation as soon as possible, even though that may mean returning for more difficult conversations as the extent of the harm becomes clear. Insurers in the UK and elsewhere are very clear about the importance of giving patients as full an explanation as possible, as soon as possible (known as ‘disclosure’) as this has been shown to reduce the likelihood of legal claims. However, many eye health professionals are not aware of the importance of disclosure – this is something that should be prioritised in training and we hope that this issue of the *Community Eye Health Journal* will go some way towards filling the gap.

## What should you say?

You should always start by apologising. Health workers may feel reluctant to apologise, as they worry that an apology implies that they are at fault. In most countries, however, it has been established that offering an apology does not mean that you are accepting legal liability for any harm that has occurred. A prompt and sincere apology will help the patient to come to terms with the adverse event, and to make the best recovery.

You should be sensitive to the needs of the patient. There needs to be enough time for a full discussion and sufficient privacy – this is not a conversation to have in a corridor. It may be advisable to have other family members present.

Patients usually want three things from an apology:

1. An explanation of what went wrong and how it happened. This has to be given in a way that the patient can understand, with a minimum of medical jargon. Often, we will not know exactly how something went wrong – there may have been a complex chain of errors leading to the event. If you don't know exactly how it happened, reassure the patient that you will personally investigate what went wrong, and that you will inform them of the results of your investigation when it is complete.
2. A plan to minimise or rectify the damage caused, and, if possible, some idea of the likely outcome. If the patient has been charged for treatment, you should reassure them that any additional treatment required as a result of the adverse event will be free.
3. How you plan to ensure that no one else is harmed in the same way.

## Why is this so important?

Worldwide, legal claims for medical errors cost millions of dollars every year. This money would be far better spent on treating patients and paying for, or training, health care workers. A sincere apology and full explanation reduces the risk of a claim for compensation. However, there are two much more important reasons:

1. We need to remember that a person has been harmed. Our ethical duty is to minimise that harm and to relieve the anxiety and distress they are suffering. Apologising, and explaining what went wrong, is an essential first step to help your patient recover.
2. By being open about errors, we make it easier to learn from them and for others to avoid them in future. It has been said that wise people learn from their own mistakes, to which I would add: even smarter people learn from other people's mistakes.

**Adverse event or medical error?**

An adverse event is an event that leads to a negative outcome for the patient. This would include irreversible harm and sight loss, but might also include an extended stay in hospital and the financial costs of additional clinic visits, even if there is no permanent injury to the patient. An adverse event does not include the expected worsening of the condition, despite adequate treatment; for example, continuing visual field loss in glaucoma, despite maximum medical therapy or vitreous haemorrhage in diabetic retinopathy, despite good laser treatment.

The dividing line between an avoidable medical error and an unavoidable adverse event is not always clear. If a patient suffers a posterior capsule rupture during cataract surgery, this is definitely an adverse event. However, was it avoidable? By some measures it is avoidable, in that the posterior capsule doesn't burst unless the surgeon catches it with an instrument. However, even the best surgeons have a capsule rupture rate of 0.5-1%. If the cataract was known to be complex, but a junior trainee was left unsupervised to carry out the operation, that would be a medical error. However, if the consultant carried out the operation, taking every possible precaution, but the capsule rupture still occurred, it is harder to see how this could have been avoided.

## Case history 1: The cataract surgeon

A cataract surgeon is performing routine cataract surgery. While she is removing the soft lens matter, the eye suddenly becomes hard and the red reflex becomes dark. The surgeon realises that there is a large choroidal haemorrhage, immediately abandons the operation, and secures the wound.

The next day the eye has a vision of hand motions – it was 6/60 pre-operatively – and the patient is not happy.

As the operating surgeon, the cataract surgeon has a duty to apologise to the patient and to provide a full explanation. The complication was not her fault, as a choroidal haemorrhage usually occurs spontaneously. Her management of the complication was correct, and she has done nothing wrong, but she has a duty to explain to the patient what went wrong and to apologise that she has made the vision worse.

The surgeon must speak to the patient as soon as possible after the operation. Assuming it was conducted under local anaesthetic, the patient will be well aware that something went wrong, as he or she will have heard the instructions to the nurses and their replies. The patient may be imagining something even worse than a choroidal haemorrhage, so the sooner the surgeon provides an explanation, the sooner the patient will be reassured. Explain what the future management will be, and reassure the patient that there is a good chance of regaining sight in the eye once the haemorrhage has settled.

## Case history 2: The eye nurse

A woman with early mild glaucoma, treated using eye drops, has cataract surgery. One month later, she attends for postoperative review and is seen by an eye nurse. Her vision is 6/9, and she is delighted. As she leaves the clinic, she asks if she should continue with the eye drops. Assuming she is referring to the glaucoma medication, the nurse says: “Yes, you must continue to use them indefinitely.”

When the woman returns for her glaucoma follow-up appointment six months later, her vision has dropped to 6/18, the intraocular pressure is 42 mmHg and the disc is deeply cupped. The eye nurse discovers that the woman had continued to use the topical steroids she was given after the cataract operation for the last six months, because she was told to “continue the drops indefinitely”.

The nurse might think, “Why should I apologise? It was the patient's mistake that they continued to use the eye drops when she should have stopped them!” However, he is the expert, not the patient. It was his responsibility to ensure that she fully understood the instructions. As the patient is not really aware of the reduced vision in the affected eye, the nurse might also think that there is no need for an explanation, which may be embarrassing for him and distressing for the patient. However, to attempt to conceal the error is dishonest, and it prevents others from learning from this error.

As a relatively junior person in the team, he should tell a more senior person what has happened. It may be best if they accompany him when he tells the patient, in case the patient asks questions that he is unable to answer.

As a result of telling the patient, and more senior staff, about the error, a new system is introduced in the clinic: postoperative drops are separated from long-term medication, and this error never occurs again. If the nurse had kept quiet, other patients could have been harmed, but being honest about the error, (mainly due to poor communication), has led to improved care.

**Figure:**
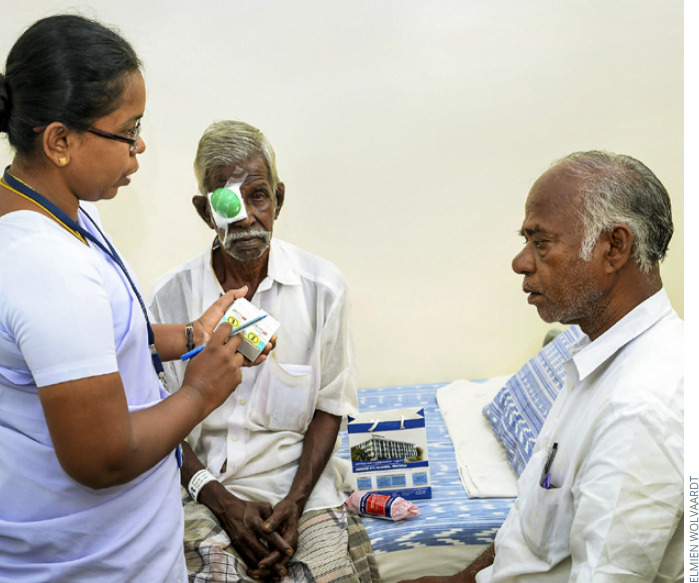
Good communication, ensuring patients understand their treatment, reduces the risk of harm; it is also critical when handling errors and adverse events.

## Case history 3: The eye clinic manager

You are the eye clinic manager. The hospital engineer tells you that an air-conditioner in the sterilisation unit is not working properly and may be blowing particles into the area where sterile instruments are placed in the instrument trays. The manager is not able to tell you how big the risk is, but she says that the air conditioner will take a week to repair. You don't want to cancel all operations for a week, as the clinic is very short of money. If there are no operations, there is no income. You assume the risk is small and don't inform the surgeons of the problem. Unfortunately, three patients operated on during the next few days develop endophthalmitis.

**“The duty of candour is not just for clinical staff. Everyone who is involved in health care has a duty to be honest and transparent about errors.”**

The duty of candour is not just for clinical staff. Everyone who is involved in health care has a duty to be honest and transparent about errors. In this case, you need to tell the surgeons about the problem with the air-conditioner, and why you decided to let the operations go ahead. It might be tempting to keep quiet and try to cover up what has happened; only you and the engineer know about the problem. However, the truth inevitably does come out, and any attempt to conceal it makes the problem far worse. It is much better to be open and honest immediately than to have the truth dragged out later.

## Conclusion

It is inevitable that things will sometimes go wrong in health care, despite our best efforts. The consequences of these adverse events can be serious, but patients will have a much better outcome if the condition is recognised promptly and managed appropriately. That starts with an open conversation that acknowledges the error and provides as full an explanation as possible of what went wrong, why it happened, and how you are going to put it right. The patient's recovery starts with your apology.
